# Overeating, caloric restriction and breast cancer risk by pathologic subtype: the EPIGEICAM study

**DOI:** 10.1038/s41598-019-39346-4

**Published:** 2019-03-07

**Authors:** Virginia Lope, Miguel Martín, Adela Castelló, Amparo Ruiz, Ana Mª Casas, José Manuel Baena-Cañada, Silvia Antolín, Manuel Ramos-Vázquez, José Ángel García-Sáenz, Montserrat Muñoz, Ana Lluch, Ana de Juan-Ferré, Carlos Jara, Pedro Sánchez-Rovira, Antonio Antón, José Ignacio Chacón, Angels Arcusa, Mª Angeles Jimeno, Susana Bezares, Jesús Vioque, Eva Carrasco, Beatriz Pérez-Gómez, Marina Pollán

**Affiliations:** 1 National Center for Epidemiology, Carlos III Institute of Health, Madrid, Spain; 2 Consortium for Biomedical Research in Epidemiology & Public Health, CIBERESP, Madrid, Spain; 3 GEICAM Spanish Breast Cancer Group, Madrid, Spain; 4 Centro de Investigación Biomédica en Red de Oncología, CIBERONC-ISCIII, Madrid, Spain; 5 Instituto de Investigación Sanitaria Gregorio Marañón, Madrid, Spain; 6 Instituto Valenciano de Oncología, Valencia, Spain; 7 Hospital Virgen del Rocío, Sevilla, Spain; 8 Hospital Puerta del Mar, Cádiz, Spain; 9 Complejo Hospitalario Universitario A Coruña, A Coruña, Spain; 10 Centro Oncológico de Galicia, A Coruña, Spain; 11 Hospital Clínico San Carlos, Madrid, Spain; 12 Hospital Clinic i Provincial, Barcelona, Spain; 13 Hospital Clínico de Valencia, Valencia, Spain; 14 Hospital Marqués de Valdecilla, Santander, Spain; 15 Fundación Hospital de Alcorcón, Madrid, Spain; 16 Complejo Hospitalario de Jaén, Jaén, Spain; 17 Hospital Universitario Miguel Servet, Zaragoza, Spain; 18 Hospital Virgen de la Salud, Toledo, Spain; 19 Consorci Sanitari de Terrassa, Barcelona, Spain; 20 Universidad Miguel Hernández, ISABIAL, Alicante, Spain

## Abstract

This study analyzes the association of excessive energy intake and caloric restriction with breast cancer (BC) risk taking into account the individual energy needs of Spanish women. We conducted a multicenter matched case-control study where 973 pairs completed lifestyle and food frequency questionnaires. Expected caloric intake was predicted from a linear regression model in controls, including calories consumed as dependent variable, basal metabolic rate as an offset and physical activity as explanatory. Overeating and caloric restriction were defined taking into account the 99% confidence interval of the predicted value. The association with BC risk, overall and by pathologic subtype, was evaluated using conditional and multinomial logistic regression models. While premenopausal women that consumed few calories (>20% below predicted) had lower BC risk (OR = 0.36; 95% CI = 0.21–0.63), postmenopausal women with an excessive intake (≥40% above predicted) showed an increased risk (OR = 2.81; 95% CI = 1.65–4.79). For every 20% increase in relative (observed/predicted) caloric intake the risk of hormone receptor positive (p-trend < 0.001) and HER2+ (p-trend = 0.015) tumours increased 13%, being this figure 7% for triple negative tumours. While high energy intake increases BC risk, caloric restriction could be protective. Moderate caloric restriction, in combination with regular physical activity, could be a good strategy for BC prevention.

## Introduction

Breast cancer is the most common malignant tumour in women worldwide. Compared to European levels, rates in Spain in 2015 are somewhere in the middle, with an age-standardised rate of 88.3 cases^1^ and 15.7 deaths^2^ per 100,000 women-years (based on the European standard).

The role of energy intake on breast cancer risk has been studied in both experimental and observational studies. According to the International Agency for Research on Cancer, there is sufficient evidence from experimental animal studies indicating that limiting body-weight gain by caloric restriction has a preventive effect on cancer of the mammary gland^3^. However, the evidence from epidemiological studies is less consistent^4–8^. Suggested biological mechanisms for the beneficial effects of caloric restriction include decreased circulating levels of growth factors, anabolic hormones, and cytokines. This in turn leads to a reduction in growth factor signaling, vascular perturbations, and inflammation. Caloric restriction is also involved in lower oxidative stress and free radical-induced DNA damage, better DNA repair processes, improved apoptosis of damaged cells, enhanced autophagy and protection from the damaging effects of toxic and genotoxic agents^9,10^.

On the other hand, high-energy intake has not been consistently associated with increased breast cancer risk in human studies^11,12^. Instead, a positive energy balance, characterized by excessive energy intake (nutritional intake) in relation to energy expenditure (physical activity)^6^, rather than high energy intake per se, may be the etiologically relevant factor in breast cancer risk. This positive balance over a prolonged period, results in excess of body fat and, consequently, contributes to increased postmenopausal breast cancer risk^12,13^. Although only a few epidemiologic studies have explored the three components of energy balance (energy intake, energy expenditure, body mass) simultaneously, all of them detected an increased risk for breast cancer among women with the least favorable energy balance profile: high-energy intake, high body mass index (BMI) and low physical activity^14–18^.

However, beyond physical activity, a more comprehensive measure of energy expenditure is the daily total energy expenditure. This measure combines three basic components: basal metabolic rate, energy expenditure through physical activity, and the metabolic response to food^19^. Basal metabolic rate is defined as the energy required for maintaining the body’s vital functions under resting conditions, and represents 45 to 70 percent of daily total energy expenditure. This rate is determined by the individual’s age, gender, body size, and body composition^19^. It would be very interesting to explore the association between excess or deficit of caloric intake and breast cancer risk taking into account the basal metabolic rate of each individual. So, the main aim of this study is to investigate the association between relative caloric intake, as a function of daily total energetic expenditure and body size, and the risk of breast cancer in Spanish women. Secondary aims include evaluating whether this association differs by menopausal status, specific risk profile, and/or tumour pathologic subtype.

## Materials and Methods

### Study population

Between 2006 and 2011, GEICAM, the Spanish Breast Cancer Group (http://www.geicam.org/), recruited 1017 women with histologically confirmed incident breast cancer being treated at 23 hospitals, in 9 Spanish regions. To be eligible for the study participants had to reside in one of the hospitals´ catchment area, be between 18–70 years old and be able to complete the epidemiological questionnaire. Each patient (case) was matched with a healthy woman (control) usually selected by the patient (friend, neighbor, co-worker…) of a similar age (±5 years) and residing in the same city. Patients´ blood-relatives were not eligible.

All procedures performed in studies involving human participants were in accordance with the ethical standards of the institutional research committees, and with the 1964 Helsinki Declaration and its later amendments or comparable ethical standards. The present study was approved by the Clinical Research Ethics Committees of the following institutions: Fundación Instituto Valenciano de Oncología; Hospital Universitario Virgen del Rocío de Sevilla; Hospital Universitario Puerta del Mar; Hospital Clínico San Carlos de Madrid; Hospital Clinic de Barcelona; Hospital Clínico Universitario de Valencia; Fundación Hospital Alcorcón; Complejo Hospitalario de Toledo; Hospital Mutua de Terrassa; Hospital Universitari de Bellvitge; Hospital General Universitario de Alicante; Hospital Virgen de los Lirios de Alcoy; Hospital Universitari de Girona Dr. Josep Trueta; Hospital Mutua de Terrassa; Hospital Universitari Arnau de Vilanova de Lleida; Fundacio d. Osona per a la Recerca i Educacio Sanitaries – FORES, and by the Regional Institutional Review Boards of Burgos and Soria, Aragon-CEICA, Galicia, Cantabria and Jaen. All participants signed an informed consent and patient information was anonymized and de-identified prior to analysis. Further details regarding the study design have been previously published^20,21^.

### Data sources and measurements

Both cases and controls completed structured epidemiological questionnaires assessing demographic and anthropometric characteristics, personal and family background, gynaecological, obstetric, medical and occupational history, and lifestyle information (smoking, physical activity, and diet). Cases completed the questionnaire within 3 months after diagnosis of breast cancer. When questions referred to a specific time period for cases (e.g., the year previous to diagnosis for physical activity or five years for dietary habits), the corresponding control was asked about the same calendar period. The questionnaires were reviewed by trained interviewers at each center, clarifying any questions participants had. Data entry and quality control were performed at the GEICAM headquarters. The accuracy of the information recorded in the database was checked by randomly selecting and reviewing 10% of the questionnaires.

Data collected for each participant included age, educational level, height and weight, menopausal status, age at menarche, number of children, age at first birth, use of hormone replacement therapy (HRT), previous diagnosis of benign breast diseases, family history of breast cancer, physical activity during the year previous to diagnosis, smoking status, and diet. Based on highest educational level achieved, participants were classified into 3 categories: no formal education - up to first grade; primary education - vocational training; and university graduate or higher. BMI was calculated based on participant´s weight one year before the interview and height.

For dietary assessment, cases and controls completed a validated semiquantitative food frequency questionnaire (FFQ) similar to the Harvard questionnaire^22^. The 117-item FFQ has been adapted to and validated in different Spanish adult populations^23^, and covered eating habits during the preceding 5 years. Patients had nine options to describe how frequently they consumed each food item, ranging from “never or less than once per month” to “six or more times per day”. Responses were converted to mean daily intake in grams by multiplying consumption frequency by the nutrient content of the portion size specified in the FFQ. Finally, total intake of each nutrient and total energy intake –in kcals/day- were computed for each participant.

From these data we also calculated level of adherence to the Western, Prudent and Mediterranean dietary patterns. Western dietary pattern (characterized by a high intake of high-fat dairy products, processed meats, refined grains, sweets, high-calorie drinks, convenience food, and sauces; and by low intakes of low-fat dairy products and whole grain) and Mediterranean dietary pattern (characterized by a high intake of fish, vegetables, legumes, boiled potatoes, fruits, olives, and vegetable oil and a low intake of juices) were already associated with breast cancer risk in a previous EpiGeicam study^20^, and therefore, possible confounders in the present study. These patterns were identified grouping the EpiGEICAM FFQ items, excluding alcoholic beverages, into 26 inter-correlated food groups that were log-transformed and centered. Subsequently, Principal Components Analysis without rotation of the variance-covariance matrix was applied. We then calculated the adherence score to Western, Prudent and Mediterranean dietary patterns as a linear combination of the weights of each food group (pattern loadings) in the previously mentioned study^20^, and the log-transformed centered food group consumption reported by the participants. Adherence scores were classified into quartiles based on controls´ score distribution.

A detailed questionnaire collected a history of physical activity from recreational, occupational, and household activities, as well as an overall assessment of physical activity considering all these domains using 5 categories: sedentary (absence of any physical activity, sitting work without physical demand and/or being under care), lightly active (mostly household activities of light intensity, occupations without physical exertion, lack of recreational activity), moderately active (manual work without handling loads, household activities of higher intensity and/or some recreational activity), active (standing or walking occupations, regular recreational physical activity), very active (vigorous occupations and/or daily practice of leisure physical activity). Due to the protective effect of physical activity on breast cancer risk previously detected in these women^21^, in the present study we decided to adjust for overall physical activity during the year previous to the breast cancer diagnosis in cases or during the year prior to interview in controls.

### Statistical analysis

Relative caloric intake was our main variable of interest as a potential predictor of breast cancer risk. Its creation consists of several steps detailed below. First, we calculated the basal metabolic rate using the predictive equations derived from meta-regression equations of 47 studies developed by Sabounchi *et al*.^24^. These equations take into account individual´s age, weight, height, gender, and race/ethnicity. An online basal metabolic rate prediction tool is also available (http://www.sdl.ise.vt.edu/tutorials.html). Next, we built a linear regression model based on data from the control group to estimate the number of calories expected, according to women’s basal metabolic rate and physical activity as follows: the amount of calories consumed by women in the control group was the dependent variable, the basal metabolic rate was included as an offset in the model and physical activity level was included as explanatory variable. From this model, we predicted the expected caloric intake per participant, and its 99% confidence interval (expected calories ±2.58 times the standard error). Afterwards, we constructed a new variable (relative caloric intake) as follows: The reference category (normal caloric intake) included participants whose caloric intake (observed) was within the range defined by the 99% confidence interval of their expected caloric intake. Overeating was defined as a relative caloric intake exceeding the upper limit (UL) of that interval (observed > UL_expected) and, in turn, undereating was defined as a relative caloric intake under the lower limit (LL) of that range interval (observed < LL_expected). Relative intake was subsequently categorized taking into account the percentiles 20 and 80 of the distribution in controls, into 5 categories: evident undereating (observed/LL_expected intake ≤0.80), moderate undereating (observed/LL_expected intake >0.80 with observed intake < LL_expected), normal intake (observed > LL_expected & observed < UL_expected), moderate overeating (observed/UL_expected intake >1 and ≤1.40) and evident overeating (observed/UL_expected intake >1.40).

Our primary aim was to evaluate the potential association between relative caloric intake and a diagnosis of histologically confirmed invasive breast cancer. Thus, the values for the main outcome variable were Case vs. Control. Our secondary aim was similar, but exploring the link between caloric intake and the different pathologic breast cancer subtypes. Thus, we created a second dependent variable based on tumour classification according to estrogen receptor (ER), progesterone receptor (PR), and the human epidermal growth factor receptor 2 (HER2) status according to a simplified version of the St Gallen international consensus^25^. Patients were classified as follows: patients with hormone receptor positive (HR+) tumours (ER+ and/or PR+, with HER2-), patients with HER2+ tumours (regardless of ER/PR status), or patients with triple negative (TN) tumours (ER-, PR-, and HER2-). Controls were the reference category.

Participants’ characteristics were described using counts and percentages for categorical variables and means and standard deviations for continuous variables. To check the significance of case-control differences we used Pearson Chi-square tests and Student’s t-tests, respectively.

Our primary aim, the potential association between relative caloric intake and breast cancer risk, was assessed using conditional logistic regression models. Linear trends were tested by entering the independent variable, relative caloric intake, as a continuous term. We adjusted for potential confounders: educational level, BMI one year prior to the interview, menopausal status (these last two variables were included in the models with the corresponding interaction term), smoking status, age at first birth (with a category of nulliparous), age at menarche, history of benign breast disease, family history of breast cancer, HRT use, physical activity, and level of adherence to Western and Mediterranean dietary patterns. Additionally, we evaluated the potential effect modification of menopause on the predictive variable by including an interaction term between relative caloric intake and menopausal status. Finally, the increase in breast cancer risk per every 20% rise in relative caloric intake was also assessed by category of other explanatory variables.

Our secondary aim, to test whether the association of interest varied across pathological subtypes, was assessed using multinomial logistic regression models with pathological subtype as the dependent variable. Models were fully adjusted as described above including also age and recruiting area. Heterogeneity of effects for the different cancer subtypes was tested using the Wald statistic.

Finally, given the matching nature of our data, we used Multiple Imputation by Chained Equations to impute missing values for overall physical activity during the previous year (8.0%), HRT use (4.9%), age at menarche (0.6%), age at first birth (4.7%), smoking (0.3%), weight (2.6%) and height (8.4%). Imputation models included these variables as well as the following confounding variables with complete information: case-control status, age, parity, menopausal status, educational level, caloric intake, and previous chronic diseases. Five imputed datasets were created using STATA´s multiple imputation procedure.

All statistical analyses were performed by using STATA/MP 14.0 software.

## Results

The global participation rate was 82% (75% in cases and 90% in controls). Of the 1017 case-control pairs recruited, 44 were excluded due to implausible caloric intake values at least in one of each member (below 750 or above 4500 calories). Thus, the current analyses are based on 973 case-control participant pairs. Compared with controls, cases had lower educational level, higher proportion of postmenopausal overweight women, slightly higher age at menarche, higher frequency of previous benign breast diseases, more relatives with breast cancer, higher caloric intake and more adherence to the Western dietary pattern. The average excess of calories consumed in relation to predicted calories (based on their physical activity and their basal metabolic rate) was 296 calories in cases and 178 calories in controls (Table 1).

**Table 1:** Participant baseline characteristics among breast cancer cases and controls.

Characteristics	Cases (n = 973)		Controls (n = 973)		p value
Age, mean (±SD)	50.4	(±9.6)	50.2	(±9.4)	0.562^a^
Educational level, n(%)					
No formal education - First grade	208	(22)	160	(16)	
Second grade - Vocational training	510	(52)	491	(51)	
University graduate	255	(26)	322	(33)	0.001^a^
Age at menarche, mean(±SD)	12.6	(±1.5)	12.4	(±1.5)	0.049^b^
Age at first birth, mean(±SD)	26.5	(±4.3)	26.2	(±4.3)	0.097^b^
Number of children, n(%)					
None	215	(22)	220	(23)	
1–2	593	(61)	587	(60)	
3–4	151	(16)	149	(15)	
>4	14	(1)	17	(2)	0.942^a^
Menopausal status, n(%)					
Premenopausal	551	(57)	513	(53)	
Posmenopausal	422	(43)	460	(47)	0.084^a^
Body mass index, Kg/m^2^, mean(±SD)					
Premenopausal	24.4	(±5.9)	24.7	(±5.9)	0.225^b^
Posmenopausal	27.3	(±8.1)	26.2	(±6.4)	0.004^b^
Hormone replacement therapy use, n(%)					
Never	864	(89)	874	(90)	
Ever	109	(11)	99	(10)	0.432^b^
Previous benign breast problems, n(%)					
No	761	(78)	796	(82)	
Yes	212	(22)	177	(18)	0.047^a^
Family history of breast cancer, n(%)					
None	728	(75)	782	(80)	
Second degree	129	(13)	105	(11)	
First degree	116	(12)	86	(9)	0.012^a^
Self-assessed physical activity last year, n(%)					
Sedentary/slightly active	365	(37)	312	(32)	
Moderately active	339	(35)	394	(41)	
Active/very active	269	(28)	267	(27)	0.126^b^
Smoking status, n(%)					
Never smoker	420	(43)	404	(42)	
Former smoker for ≥6 mo.	258	(27)	254	(26)	
Smoker or former smoker for <6 mo.	295	(30)	315	(32)	0.287^b^
Caloric intake (Kcal/day), mean(±SD)	1990.2	(±615.1)	1897.2	(±628.4)	0.001^a^
Basal metabolic rate (Kcal/day), mean(±SD)	1316.2	(±137.6)	1309.8	(±122.8)	0.263^b^
Predicted calories (Kcal/day), mean(±SD)	1694.5	(±305.2)	1718.8	(±291.1)	0.072^a^
Mediterranean dietary pattern score^c^, n(%)					
<5.0812	254	(26)	243	(25)	
5.0812–6.0047	250	(26)	243	(25)	
6.0048–6.8686	263	(27)	244	(25)	
>6.8686	206	(21)	243	(25)	0.250^a^
Western dietary pattern score^c^, n(%)					
<4.104	208	(22)	243	(25)	
4.104–5.150	216	(22)	243	(25)	
5.151–6.197	254	(26)	244	(25)	
>6.197	295	(30)	243	(25)	0.023^a^
Tumour subtype^d^, n(%)					
HR+	653	(67)			
HER2+	199	(21)			
TN	119	(12)			

Abbreviations: SD = standard deviation; HR+= hormone receptor positive tumours (ER+ and/or PR + , with HER2−); HER2+= human epidermal growth factor receptor 2 positive tumours; TN = triple negative tumours.

^a^p value resulting from Pearson Chi-Square test (variables with no missing values).

^b^p value resulting from conditional logistic regression models (variables with imputed values).

^c^According to the quartiles in the control group.

^d^Two breast cancer cases could not be classified.

Table 2 shows the association between caloric consumption above or below predicted values and breast cancer risk, both overall and by menopausal status, including a test for interaction between relative caloric consumption and menopausal status. In general, women who consumed substantially fewer calories than their corresponding predicted calories (a deficit greater than 20%) had lower breast cancer risk (OR = 0.52; 95% CI = 0.35–0.78). This association held only for premenopausal women (p for heterogeneity = 0.001). Women who consumed more calories than predicted (up to 40% more) had an increased risk of breast cancer (OR = 1.38; 95% CI = 1.00–1.90), However, women who exceeded the predicted intake of calories substantially (40% or more the predicted value) had almost twice the risk compared to those whose caloric intake was within the suitable levels (OR = 1.92; 95% CI = 1.31–2.82). Such excess risk was concentrated among postmenopausal women (OR = 2.81; 95% CI = 1.65–4.79) rather than in premenopausal women (p for heterogeneity = 0.001). When we analyzed breast cancer risk for every 20% increase in relative caloric intake we observed a positive and statistically significant trend (OR_per_ 20% = 1.14; 95% CI = 1.06–1.22). No differences were seen between pre and postmenopausal women (*p*-heterogeneity = 0.734).

**Table 2:** Multivariate analysis of caloric intake above or below predicted calories and breast cancer risk by menopausal status.

	All women (n = 1946)					Premenopausal women (n = 1064)					Postmenopausal women (n = 882)					p-het
	controls	cases	OR^a^	(95% CI)	p value	controls	cases	OR^a^	(95% CI)	p value	controls	cases	OR^a^	(95% CI)	p value	
	(n = 973)	(n = 973)				(n = 513)	(n = 551)				(n = 460)	(n = 422)				
Relative caloric intake																
More than 20% below predicted calories	162	105	0.52	(0.35–0.78)	0.001	92	51	0.36	(0.21–0.63)	<0.001	70	54	0.77	(0.44–1.35)	0.365	0.001
Up to 20% below predicted calories	180	165	0.93	(0.66–1.31)	0.685	100	97	0.77	(0.49–1.24)	0.283	80	68	1.14	(0.68–1.92)	0.624	0.503
Within range of predicted calories (ref)	152	136	1.00			64	77	1.00			88	59	1.00			
Up to 40% above predicted calories	301	330	1.38	(1.00–1.90)	0.048	156	195	1.16	(0.75–1.81)	0.498	145	135	1.55	(0.98–2.47)	0.062	0.144
More than 40% above predicted calories	178	237	1.92	(1.31–2.82)	0.001	101	131	1.40	(0.85–2.31)	0.182	77	106	2.81	(1.65–4.79)	<0.001	0.001
*Trend per 20% increase over the predicted range*			*1.14*	*(1.06*–*1.22)*	<*0.001*			*1.13*	*(1.04*–*1.22)*	*0.003*			*1.15*	*(1.05*–*1.25)*	*0.002*	*0.734*

Abbreviations: OR = odds ratio; CI = confidence interval; p = p value; p-het = p value for heterogeneity.

^a^Adjusted for educational level, body mass index one-year before the interview, smoking status, age at menarche, age at first birth (with a category of nulliparous), previous benign breast problems, family history of breast cancer, hormone therapy use, recent physical activity, level of adherence to Mediterranean and Western dietary patterns, and menopausal status (all women). Adjusted for age and hospital by design.

Next, we examined the association between caloric intake and breast cancer risk by pathological subtype (Table 3). There was a statistically significant positive trend for HR+ and HER2 +tumours, with a 13% increased breast cancer risk for every 20% increase in relative caloric consumption. Women reporting substantial caloric deficits (>20% below predicted values) showed a decreased risk of HR+ tumours (OR = 0.60; 95% CI = 0.40–0.91), whereas women consuming over 40% of predicted calories had a higher risk of HR+ (OR = 1.82; 95% CI = 1.22–2.70) and HER2+ (OR = 2.05; 95% CI = 1.11–3.76) tumours. A similar trend was observed for TN tumours, although the associations failed to attain statistical significance probably due to the limited number of cases.

**Table 3:** Multivariate logistic analysis of relative caloric intake and breast cancer risk by pathological subtype^a^.

		controls	HR + (n = 653)				HER2 + (n = 199)				TN (n = 119)				p-het
		(n = 971)	cases	OR^b^	(95% CI)	p value	cases	OR^b^	(95% CI)	p value	cases	OR^b^	(95% CI)	p value	
Relative caloric intake															
More than 20% below predicted calories		162	69	0.60	(0.40–0.91)	0.015	21	0.60	(0.31–1.14)	0.119	15	0.55	(0.25–1.22)	0.142	0.980
Up to 20% below predicted calories		180	110	1.03	(0.72–1.48)	0.862	38	1.27	(0.73–2.19)	0.398	16	0.85	(0.40–1.78)	0.658	0.631
Within range of predicted calories (ref)		152	92	1.00			28	1.00			16	1.00			
Up to 40% above predicted calories		299	218	1.34	(0.96–1.88)	0.083	65	1.44	(0.86–2.42)	0.164	46	1.69	(0.89–3.22)	0.109	0.782
More than 40% above predicted calories		178	164	1.82	(1.22–2.70)	0.003	47	2.05	(1.11–3.76)	0.021	26	1.89	(0.88–4.07)	0.102	0.933
*Trend per 20% increase over the predicted range*				*1.13*	*(1.06*–*1.21)*	<*0.001*		*1.13*	*(1.03*–*1.25)*	*0.015*		*1.07*	*(0.94*–*1.21)*	*0.318*	*0.670*

Abbreviations: HR+= hormone receptor positive tumours (ER+ and/or PR+, with HER2-); HER2+= human epidermal growth factor receptor 2 positive tumours; TN = triple negative tumours; OR = odds ratio; CI = confidence interval; p = p value; p-het = p value for heterogeneity.

^a^Two breast cancer cases could not be classified.

^b^Adjusted for age, hospital, educational level, body mass index one-year before the interview, smoking status, age at menarche, age at first birth (with a category of nulliparous), menopausal status, previous benign breast problems, family history of breast cancer, hormone therapy use, recent physical activity, and level of adherence to Mediterranean and Western dietary patterns.

**Figure 1:**
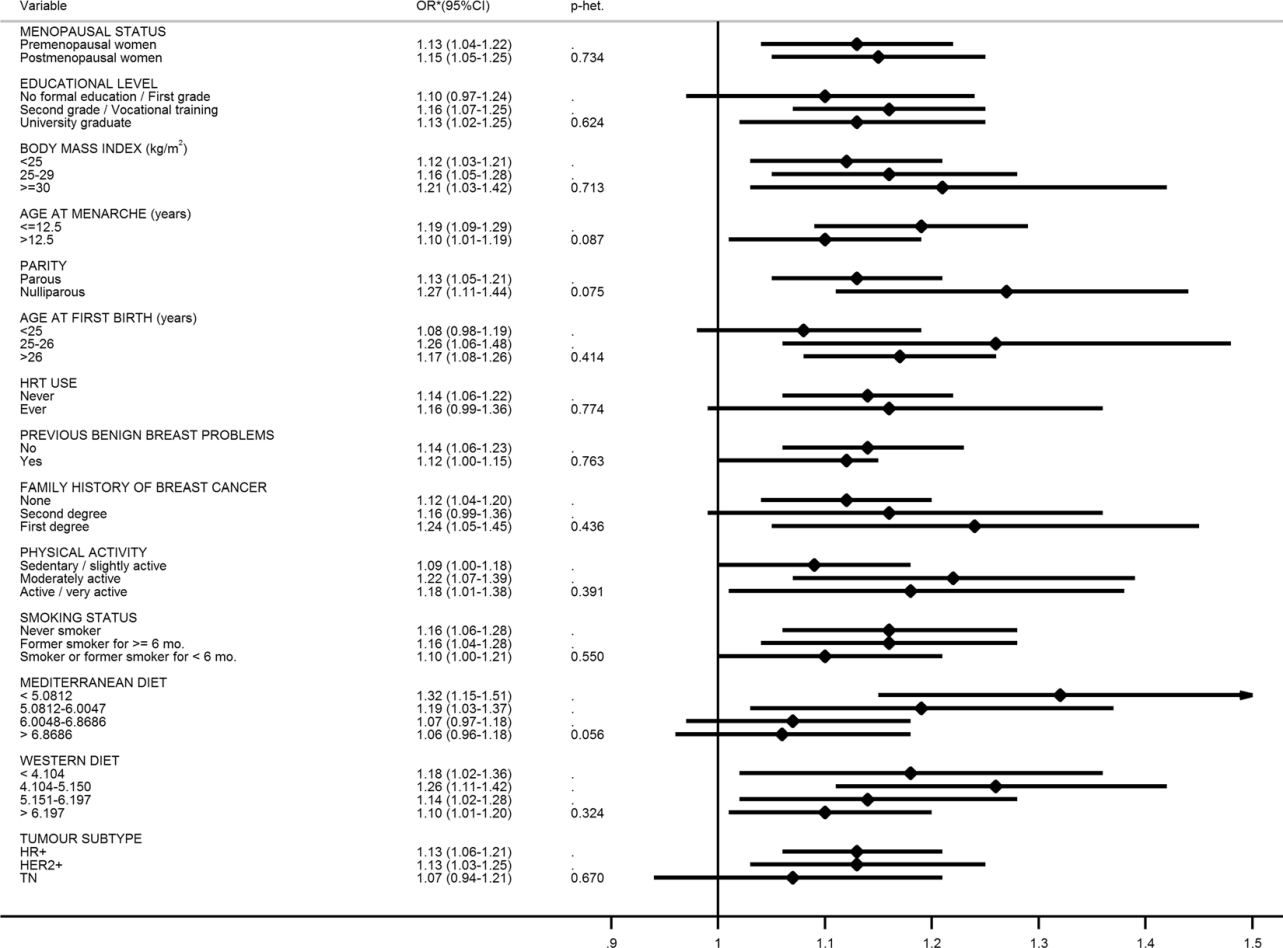
Breast cancer risk for every 20% increase in relative calorie consumption according to women characteristics. *Adjusted for menopausal status, educational level, BMI one-year before the interview, smoking status, age at first birth, age at menarche, previous benign breast problems, family history of breast cancer, hormone replacement therapy use, self-assessed physical activity during the last year, Mediterranean dietary pattern and Western dietary pattern.

Figure 1 depicts the breast cancer risk for every 20% increase in relative caloric consumption per category of the other explanatory variables. The effect was very consistent across all strata. However, it is worth noting the particularly increased risk observed in women with low adherence to the Mediterranean dietary pattern (OR = 1.32; 95% CI = 1.15–1.51; p-heterogeneity = 0.056), as well as in nulliparous women (OR = 1.27; 95% CI = 1.11–1.44; p-heterogeneity = 0.075).

## Discussion

The present study analyzes the association between breast cancer risk and women’s relative caloric intake (actual/predicted intake) according to participants’ physical activity and basal metabolic rate. Our results suggest that restricted caloric intake offers a protective effect against breast cancer, especially in premenopausal women, whereas excessive caloric consumption increases breast cancer risk. The latter effect is observed mainly among postmenopausal women and women reporting low adherence to the Mediterranean diet. A positive association between relative caloric intake and breast cancer was observed in all pathologic subtypes, though the association failed to reach statistical significance among women with TN tumours due, most likely, to a limited number of cases.

This study has several strengths. First, this is a multicenter study carried out in 9 Spanish regions located throughout the second largest country in Europe. Second, to our knowledge, this is the first epidemiological study to date that explores the effect of caloric deficit or excessive caloric consumption according to the individual energy requirements of each woman based on her basal metabolic rate and physical activity performed. Previous studies have examined the joint effect of energy intake, physical activity, and body size; however, their analyses were stratified by those three components^14–18^. Third, because physiological pathways and metabolic effects of calories vary by source (i.e., calories from proteins, fats, carbohydrates…) we adjusted our models for quartile levels of adherence to the Mediterranean and Western dietary patterns, both associated with breast cancer risk and previously identified among our participants^20^. All the possible combinations of these two variables allowed us to capture (and adjust for) the broad diversity of food consumption patterns found across Spain. Fourth, regarding breast cancer subtypes, as far as we are aware of, this is the first study exploring the association between caloric intake and breast cancer risk considering HER2 status separately. Finally, the large sample of premenopausal participants allowed for a fully adjusted exploration of the effect of caloric consumption on this group.

Several limitations should be considered when interpreting our findings. First, the explanatory variables of interest are based on self-reported data, thus subject to recall bias. In particular, even though the FFQ used showed a satisfactory validity and reproducibility^23^, there is evidence that overweight or obese individuals tend to underestimate their weight and caloric intake and to overestimate their physical activity^26^. To minimize this bias, we performed sensitivity analyses to test whether the effect of caloric intake was consistent across four categories of BMI (<20, 20–25, 25–30 and >30 Kg/m^2^). The resulting associations were very similar to those reported here, with no evidence of heterogeneity (data not shown). Therefore, we believe that this potential bias, is very likely non-differential, and thus, our reported associations would, in any case, underestimate the real ones. Further, although we collected very detailed information on recreational physical activity^21^ we forewent this information in favor of categories of overall physical activity to estimate the expected caloric consumption. We made this decision because leisure physical activity does not realistically capture the amount of exercise performed by women in this context, especially in the case of less educated women^27^. However, when sensitivity analyses were performed with the subgroup of women with complete information on metabolic equivalents (MET-h/week) from leisure physical activities, the results were no different from those reported here (data not shown). On the other hand, the fact of having selected friends, partners or not blood-relatives as controls of breast cancer cases could entail a greater similarity in lifestyles, including dietary habits among cases and controls. However, if this bias exists, it would imply an underestimation of the effects studied, and so, the associations reported here would underestimate the real ones. Being a case-control study, another limitation to take into account is that all information (dietary habits, physical activity, etc) in cases was collected after diagnosis. This may have caused differential recall between cases and controls that could have skewed the results. However, this differential bias could be minimized because women were not aware of the specific hypothesis we investigated and we used a well-structured and validated instrument for dietary assessment with a wide temporal window (5 years prior to the diagnosis of the corresponding case) thus expecting similar misclassification errors in cases and controls. Regarding pathologic subtypes, we had an insufficient number of cases with TN tumours to achieve statistical power to test heterogeneity, since TN tumours are relatively uncommon in our patient population^28^. Finally, even though most established risk factors were taken into account, residual confounding cannot be ruled out.

Our results show that women who consumed fewer than 80% of the calories expected based on their physical activity and basal metabolic rate, had lower breast cancer risk. There is sufficient evidence based on animal models that limiting body weight gain by caloric restriction prevents cancer of the mammary gland^3^. Some observational epidemiological studies provide additional evidence that caloric restriction provides humans certain protection against cancer^4,8,9^. In fact, recent clinical intervention trials have demonstrated that some metabolic and molecular adaptations observed in experimental animals also take place in humans in response to caloric restriction^29^. Short-term studies with overweight and obese subjects have shown that caloric restriction may reduce circulating concentrations of inflammatory markers such as C-reactive protein, tumour necrosis factor-α and interleukin-6, improve insulin sensitivity, increase serum concentrations of insulin-like growth factor binding protein (IGFBP)-1 and IGFBP-2, increase circulating levels of adiponectin, and reduce leptin and the leptin-to-adiponectin ratio^9,30^. In addition, caloric restriction affects multiple processes involved in the pathogenesis of cancer including a reduction of oxidative stress and free radical-induced DNA damage, an enhancement of DNA repair processes, an increase in removal of damaged cells through apoptosis, an enhancement in autophagy and protection from the damaging effects of toxic agents^9,10^.

The protective effect of caloric restriction has been observed in both our premenopausal and postmenopausal participants. Previous observational studies have shown that weight loss through caloric restriction reduces the risk of postmenopausal breast cancer^4,30^. In premenopausal participants of a randomized trial, Harvie and colleagues observed that both intermittent and continuous caloric restriction during 6 months resulted in weight loss in 107 overweight or obese women together with improvements in several cancer risk markers^31^. Among the metabolic effects of this voluntary weight loss, several studies have demonstrated a substantial reduction in biomarkers of estrogen stimulation and inflammation, among both premenopausal and postmenopausal women^32^. However, we observed that postmenopausal women in our study had to consume substantially fewer calories than predicted to reduce their breast cancer risk. Thus, we conclude that postmenopausal women would need a stricter hypocaloric diet to reduce their body fat and, hence, their estrogen exposure through aromatization of adrenal androgens.

Although most case-control and prospective studies have found a positive association between high energy intake and breast cancer risk, others have not^11,12^. Excessive caloric intake results in weight gain and body fatness which, in turns, is strongly associated with postmenopausal breast cancer risk. However, greater body fatness before the menopause seems to protect against premenopausal breast cancer^12,13^. To take into account the possible differential effect of obesity in pre and postmenopausal women, all models were adjusted by the interaction term between both variables. Nevertheless, in our study, the association between obesity and breast cancer was not significant among premenopausal women (OR = 0.98, IC(95%) = 0.95–1.02). Interestingly, although breast cancer risk associated with excessive caloric intake was significantly higher among postmenopausal women, the risk for every 20% increase in relative caloric consumption was practically the same in both groups. It should be borne in mind that body fatness is the result of maintained energy disbalance in the past, and from that perspective, overeating in premenopausal women will translate into higher BMI in the future, possibly after menopause. Taking this consideration into account, our results do not contradict, but are in agreement with what is already known based on BMI. From a public health point of view, they emphasize the importance of reducing excessive caloric intake before menopause. On the other hand, because total caloric intake is highly related to body size and physical activity, it is difficult to assess the independent effect of caloric intake on breast cancer risk. Two previous cohort studies found that women with the most unfavorable balance profile (i.e., high energy intake, high BMI, and low physical activity) had increased premenopausal^17^ and postmenopausal^14^ breast cancer risk. Results further confirmed by two case-control studies^16,18^. Whereas Mallin and co-authors observed that the profile characterized by high energy intake and low physical activity was associated with a higher breast cancer risk among postmenopausal women^16^, Zhang and colleagues found a 60%-70% increased risk associated with high energy intake only among premenopausal women^18^.

In our study, relative caloric intake seemed to have a similar effect on the risk of all pathologic subtypes of breast cancer, suggesting that there must be biological mechanisms, other than those related to the hormonal pathway, influencing this association. Zhang *et al*., in a case control study of women enrolled in the Breast Cancer Family Registry, found that the association between high energy intake and breast cancer risk was only significant in women with HR+ tumours^18^. In addition, some experimental studies with mice have shown that caloric restriction may reduce the risk of HER2+ breast cancer^33^, decreases metastatic burden in TN breast cancer patients^34^, or slow tumour growth regardless of tumour subtype^35^.

The breast cancer risk associated with excessive caloric consumption was higher in women with low adherence to the Mediterranean dietary pattern. This result is in line with the qualitative approach by Lucan and colleagues^36^. According to this approach, what contributes to obesity and related diseases is not the number of calories in specific foods but rather the amount and type of carbohydrates these foods contain. Foods that are highly processed, and mostly comprised of rapidly absorbable sugars and starches, may be of greatest concern. Such carbohydrates, not typical of the Mediterranean diet, may induce neurohormonal alterations that lead to “eating more” and “moving less”^36^. In contrast, Bray *et al*.^37^ found that, given equal numbers of extra calories, weight gain was lower with a low protein diet compared with a normal protein diet. Whereas calories alone contributed to body fat increase, protein intake contributed to changes in energy expenditure and storage of lean body mass, but not to an increase in body fat^37^.

In summary, we found that women with a high relative caloric intake, i.e., above the expected intake according to physical activity and body size, presented increased breast cancer risk, with caloric restriction showing a protective effect. This association was observed in both premenopausal and postmenopausal women and across all pathologic breast cancer subtypes. Given that the prevalence of sedentarism is increasing in Spain (44% reported never exercised or played sports in the 2013 Eurobarometer Survey)^38^, that the prevalence of overweight and obese women has also increased progressively (54.6% in 2014)^39^, and that the consumption and variety of fast food has spread widely in recent years in Spanish households^40^, individual and public health strategies should be implemented especially in high-risk women. Moderate caloric restriction in combination with regular physical activity could have beneficial effects on health. Future prospective studies are needed to confirm these results in other populations and to elucidate the mechanisms underlying them.

## Data Availability

The datasets on which the present analyses are based are not publicly available, owing to incompatibility with the regulations of the different ethics committees in the contributing study centers. However, these datasets may be available from GEICAM upon request, provided that all committees accept it.
